# Omentin-1 is associated with atrial fibrillation in patients with cardiac valve disease

**DOI:** 10.1186/s12872-020-01478-1

**Published:** 2020-05-06

**Authors:** Yubin Chen, Fen Liu, Fei Han, Lizhi Lv, Can-e Tang, Zhongshang Xie, Fanyan Luo

**Affiliations:** 1 Department of Cardiac Surgery, Xiangya Hospital, Central South University, Changsha, 410008 Hunan China; 2 The Institute of Medical Science Research, Xiangya Hospital, Central South University, Changsha, 410008 Hunan China

**Keywords:** Epicardial adipose tissue (EAT), Atrial fibrillation, Atrial fibrosis, Omentin-1

## Abstract

**Background:**

Epicardial adipose tissue (EAT) remodeling and adipocytokines are associated with structural remodeling in atrial fibrillation (AF). However, the role of omentin-1, a novel adipocytokine, in structural remodeling remains unknown.

**Methods:**

Hematoxylin and eosin (H&E) and Masson’s trichrome stains were used to investigate the histology of EAT and right atrial appendages. The expression levels of adipocytokines in these human samples were determined by immunohistochemical assay and western blotting. Models of transforming growth factor (TGF)-β1-induced activation of cardiac fibroblasts (CFs) and TGF-β1-induced endothelial-mesenchymal transition (EndMT) of human umbilical vein endothelial cell (HUVEC) were established to explore roles of omentin-1 in these processes. To determine changes in adipocytokines secretion under hypoxia conditions, adipocytes were treated with 5% O_2_ and 95% N_2_, and then CFs and HUVECs were co-cultured with the conditioned medium of adipocytes to determine the effects of hypoxia-treated adipocytes on these cells.

**Results:**

Expression of omentin-1 was downregulated in the EAT and right atrial appendages from patients with AF compared to samples from patients without AF, while the TGF-β1 level was upregulated in EAT from patients with AF. EAT from patients with AF exhibited adipocyte hypertrophy and severe interstitial fibrosis. Omentin-1 inhibited TGF-β1-induced CF activation and reversed TGF-β1-induced HUVEC EndMT. Adipocytes treated with hypoxia exhibited downregulation of omentin-1 and partly activated CFs.

**Conclusions:**

This study demonstrated that omentin-1 was an antifibrotic adipocytokine and was downregulated in patients with AF, which was partly mediated by hypoxia.

## Background

Atrial fibrillation (AF), which is the most common type of cardiac arrhythmia, affects approximately 33.5 million individuals in 2010 worldwide [1]. The primary causes of AF are structural and electrical remodeling in the myocardium. Excessive extracellular matrix (ECM) production and accumulation in the atrium, termed atrial fibrosis, is the hallmark of structural remodeling. Severe atrial fibrosis could cause paroxysmal AF to become permanent AF and even reduce the effectiveness of antiarrhythmic therapy [2]. Cardiac fibroblasts (CFs) play a pivotal role in atrial fibrosis. In healthy adults, CFs usually remain in an inactive state. However, under pathological conditions, these cells proliferate rapidly and differentiate to myofibroblasts that expressed alpha smooth muscle actin (α-SMA), which then synthesize and secrete abundant ECM components including collagen-1 (COL1) and collagen-3 (COL3) [3]. Simultaneously, some cell lineages including endothelial cells and monocytes differentiate into CFs, contributing to atrial fibrosis [4].

Epicardial adipose tissue (EAT), which surrounds the myocardium and coronary artery without fascia separation, is a secretory organ that exerts paracrine [5] and vasocrine [6] effects on the myocardium and coronary artery. EAT structure and secretion phenotype change in cardiovascular diseases. Mahajan et al. demonstrated that EAT was more diffusely distributed in an obese sheep model with AF vulnerability [7]. Tomasz et al. reported that EAT of patients with coronary artery disease had higher expression levels of interleukin (IL)-1, IL-6, monocyte chemoattractant protein 1 and tumor necrosis factor alpha than subcutaneous adipose tissue [8]. However, the distribution of EAT was limited in lean sheep [7] and the tissue also secreted some beneficial adipocytokines such as adiponectin and omentin-1 [9]. As a novel adipocytokine that is secreted mainly from visceral adipose tissue, omentin-1 can suppress the proliferation, migration, and ECM production of vascular smooth muscle cells [10], ameliorate arterial calcification through the receptor activator of NF-κB (RANK) signaling pathway [11], and be used to predict the development of coronary collateral circulation as well as the occurrence and development of coronary heart disease [12, 13].

Recently, Tao et al. reported that the serum level of omentin-1 was inversely correlated with AF development and atrial structural remodeling [14]. However, the exact mechanism of how omentin-1 mediates atrial fibrosis is still unknown. In this study, we analyzed EAT structure, and investigated the expression levels of omentin-1 and transforming growth factor (TGF)-β1 in EAT and right atrial appendages. Then, models of TGF-β1-induced activation of CFs and TGF-β1-induced endothelial-mesenchymal transition (EndMT) of human umbilical vein endothelial cell (HUVEC) were established to explore the roles of omentin-1 in these processes. Finally, investigation has been conducted to figure out what caused the change in the adipocyte-secretion phenotype.

## Methods

### Collection of humans EAT and right atrial appendages

The study was approved by the Ethic Committee of Xiangya Hospital Central South University. Human EAT and right atrial appendages were obtained from patients who underwent cardiac valve surgery in the Department of Cardiac Surgery, Xiangya Hospital, Central South University. EAT samples were obtained from the root of aorta to avoid bleeding and cardiac injury. Right atrial appendages were derived from patients who underwent superior vena cave intubation via excision of right atrial appendages before extracorporeal circulation. Patients were diagnosed with AF according to the guidelines of the American Heart Association [15]. Patients were excluded based on the following criteria: history of cardiac surgery; age < 18 years; and presence of a tumor, infectious endocarditis, or systemic inflammatory disease. All samples were stored at − 80 °C. Right atrial appendages (*n* = 8) were used for western blot analysis. Partly paired EAT (*n* = 40) and right atrial appendages (*n* = 31) were used for hematoxylin and eosin (H&E) staining, Masson’s trichrome staining, and immunohistochemistry. Samples were collected from 40 patients. In detail, 31 of them underwent superior vena cave intubation via excision of right atrial appendages while 9 of them underwent superior vena cave intubation via direct incision of right atrium. In general, right atrial appendages and EAT could be obtained from these 31 patients, but for the remaining 9 patients, only EAT could be obtained.

### Histology of EAT and right atrial appendages

Formalin-fixed, paraffin-embedded sections (4 μm) were deparaffinized and subjected to Masson’s trichrome staining and H&E staining. A light microscope (Leica DM5000 B) was used to obtain images (5 fields for each section). The adipocyte size was calculated as the ratio of total adipocyte size/adipocyte number, while the interstitial fibrosis area ratio was calculated using the interstitial fibrosis area (stained blue)/total adipose area. Image-Pro Plus 6.0 software was used to analyze adipocyte size in images of tissues stained with H&E, and interstitial fibrosis area ratio was determined using images of tissue stained with Masson’s trichrome.

### Immunohistochemical assay

Formalin-fixed, paraffin-embedded sections (4 μm) were deparaffinized and hydrated. After antigen retrieval, sections were incubated with primary antibodies against omentin-1 (1:200 dilution, GTX32687, GeneTex) and TGF-β1 (1200 dilution, ab92486, abcam). Then, sections were stained using a detection kit (Beijing Zhongshanjinqiao Biotechnology Co., Ltd.) and hematoxylin (Beijing Zhongshanjinqiao Biotechnology Co., Ltd.). Images were obtained with a light microscope (5 fields for each section). The mean integrated optical density (IOD) was analyzed using Image-Pro Plus 6.0 software.

### Western blot analysis

Tissues were homogenized in radioimmunoprecipitation assay (RIPA) buffer (WB3100, NCM Biotech) containing phenylmethylsulphonyl fluoride (PMSF G2008, Servicebio) and phosphatase inhibitors (B15001, Bimake) using TissueLyser (Qiagen). Cells were lysed in RIPA buffer containing PMSF and phosphatase inhibitors. The suspension was centrifuged at 12,000×g at 4 °C for 30 min, and then the supernatant was obtained. Protein concentration was determined by the bicinchoninic acid (BCA) assay (23,227, ThermoFisher Scientific). Next, 20 μg of protein was separated on a 10% Bis-Tris gel, then transferred to a polyvinylidene.

fluoride (PVDF) membrane (IPVH00010, Millipore), blocked in 5% bovine serum albumin at 25 °C for 2 h, incubated overnight at 4 °C with primary antibodies against the following proteins: glyceraldehyde 3-phosphate dehydrogenase (GAPDH) (1:5000 dilution, T0004, Affinity), tubulin (1:8000 dilution, ab6046, abcam), alpha smooth muscle actin (α-SMA) (1:1000 dilution, ab5694, abcam), collagen-1(COL1) (1:800 dilution, ab34710, abcam), collagen-3 (COL3) (1:800 dilution, ab7778, abcam), pSmad2 Ser465/Ser467 (1:800 dilution, #3108, CST), pSmad3 Ser423/425 (1:800 dilution, #9520, CST), SMAD2 (1:1000 dilution, #5339, CST), SMAD3 (1:1000 dilution, #9523, CST), vimentin (1:1000 dilution, 14–9897-82, Invitrogen), VE-Cadherin (VE-Cad, 1:800 dilution, #5741, CST), omentin-1 (1:1000 dilution, GTX32687, GeneTex). Subsequently, the membrane was incubated with corresponding horseradish peroxidase (HRP)-conjugated secondary antibodies (1:8000 dilution, #S0001 #S0002, Affinity) at 25 °C for 90 min, and finally observed with Enhanced Chemiluminescent (p10100, NCM Biotech) on the ChemiDoc XRS Plus (Bio-Rad). Relative protein expression levels were analyzed using Image Lab 3.0 software.

### Cell culture and treatment

Primary rat CFs purchased from Sciencell (R6300) were cultured in Dulbecco’s modified Eagle’s medium (DMEM Hyclone) containing 10% fetal bovine serum (FBS), 1% penicillin-streptomycin, and 1% fibroblast-growth-supplement-2 (Cat.No.2382, Sciencell). HUVECs were cultured in DMEM containing 10% FBS and 1% penicillin-streptomycin. Cells at passages 3–8 were used.

For the treatments, cells at confluency were starved for 24 h in serum-free DMEM, and then the medium was replaced with FBS-containing DMEM. The cells were then incubated for 24 h with recombinant human TGF-β1 (100–21-10, PeproTech) and recombinant human omentin-1 (9137-IN-050, R&D) at the following concentrations: for both CFs and HUVECs: control, 10 ng/mL TGF-β1, 10 ng/mL TGF-β1 + 50 ng/mL omentin-1, 10 ng/mL TGF-β1 + 100 ng/mL omentin-1, 10 ng/mL TGF-β1 + 150 ng/mL omentin-1, 10 ng/mL TGF-β1 + 200 ng/mL omentin-1. After incubation, total protein and RNA were extracted from cells for further analyses.

### 3 T3-L1 cells culture, differentiation and treatments

3 T3-L1 cells were purchased from Beijing Zhongshanjinqiao Biotechnology Co., Ltd., and cultured in DMEM containing 10% FBS and 1% penicillin-streptomycin. To induce cell differentiation, the following 3 media were used: M1, comprising DMEM with 10% FBS and 1% penicillin-streptomycin; M2, comprising M1, 1.5 μg/mL insulin (91077C, sigma), 0.5 mM 3-isobutyl-methylxanthine (IBMX) (I5879, sigma), 1 μM dexamethasone (D4902, sigma), and 2 mM rosiglitazone (R2408, sigma); M3, comprising M1 and 1.5 μg/mL insulin. First, cells were cultured in M1. Once cells reached confluency, the medium was replaced with M2 and defined as differentiation day 0. On day 2, the medium was replaced with M3 and then changed to M2 again on day 4. From day 6 onward, cells were maintained with M3, with medium changes every 2 days [16]. Cells at days10–12 were considered as adipocytes for use in the following experiments.

Adipocytes were starved for 24 h in serum-free DMEM, and then the medium was replaced with M1. Adipocytes of the normoxia group were cultured under 5% CO_2_ in a 37 °C incubator, whereas adipocytes of the hypoxia group were cultured under 5% O_2_ + 95% N_2_ in a 37 °C incubator. Both groups were cultured for 12, 24, and 36 h. Conditioned medium (CM) was collected and total protein and RNA were extracted from cells for further analyses.

### Quantitative reverse transcription-polymerase chain reaction (RT-qPCR)

Total RNA was extracted using TRIzol reagent (15,596,018, Invitrogen) and reverse transcribed using HiScript II QRT SuperMix (R223–01, Vazyme). The RT-qPCR was performed on a ViiA 7 system (Applied Biosystems) using the All-in-One qPCR Mix (GeneCopoeia) for 40 cycles. GAPDH was used as a control for normalization. The primers used in this study are shown in Supplementary Table 1.

### Immunofluorescence

Eight hundred starved cells were seeded into each well of a 96-wells plate, and treated for 24 h with recombinant human TGF-β1 and recombinant human omentin-1 at the concentration mentioned above. After incubation, cells were rinsed 3 times with phosphate-buffered saline (PBS), fixed with 4% paraformaldehyde for 20 min, permeated with 0.5% Triton-X100 for 15 min, blocked with normal goat serum for 60 min, and incubated overnight at 4 °C with primary antibodies against the following proteins: α-SMA (1:200, dilution), vimentin (1:200, dilution), and VE-Cad (1:100, dilution). This was followed by incubation for 40 min at 37 °C with corresponding secondary antibodies: Alexa Fluor 488 goat-anti-rabbit IgG H&L (1:500 dilution, ab150077, Abcam) and Alexa Fluor 594 donkey-anti-mouse IgG (H + L) (1:500 dilution, 715–585-150, Jackson Laboratories). Thereafter, cells were stained for 5 min with 4′,6-diamidino-2-phenylindole (DAPI, 1:1000 dilution, 564,907, BD Pharmingen), and images were then acquired using the PerkinElmer Operetta CLS system. The fluorescence intensity was analyzed using Harmony 3.5 software.

### Scratch assay

Cells were seeded onto 6-well plates at 2 × 10^5^ cells/well and starved for 24 h with serum-free medium. Then, the cell layer on the surface of each well was gently scratched using 10-μL pipette tips. The wells were rinsed 3 times with PBS to remove cell debris, and then medium containing 2% FBS with recombinant human TGF-β1 and recombinant human omentin-1 (concentration as mentioned above) was added. Images were obtained at 0, 12, and 24 h using a light microscope.

### Cell proliferation assay

Cell proliferation was measured using the Cell Counting Kit-8 (C008, 7Sea Biotech) according to the manufacturer’s recommended procedures. In brief, 1000 serum-starved cells were seeded onto 96-well plates and treated with recombinant human TGF-β1 and recombinant human omentin-1 at the concentrations mentioned above. The optical density values were measured at 0, 12, 24, 36, and 48 h using a spectrophotometer.

### Enzyme-linked immunosorbent assay

The omentin-1 concentration in the CM was measured using an enzyme-linked immunosorbent assay kit (RayBiotech EIAM-OME-1) according to the manufacturer’s recommended procedures.

### Statistical analysis

Data are presented as the means ± standard error of mean (SEM). Continuous data were compared using the unpaired Student t-test and two-way analysis of variance. The unpaired Student’s t-test, Chi-square test, and Mann-Whitney U test were used to compare differences between the patients. Statistical analyses were performed using SPSS 19 software. *P* values < 0.05 were considered as being statistically significant.

## Results

### EAT structure, and expression levels of omentin-1 and TGF-β1 in human samples

The patients’ baseline data are shown in Table 1. The left atrial dimension (LAD) and right atrial dimension (RAD) of the patients with AF were larger than those without AF, which were consequences of structural remodeling. Interestingly, we found that the high-density lipoprotein (HDL) content was downregulated in patients with AF. Table 2 displays the surgery of patients in each group. Patients who underwent mitral valve replacement (MVR) + tricuspid valvuloplasty (TVP) in AF group were more than those in nAF group (47.9% vs. 12.5%, *p* < 0.001) while patients who underwent aortic valve replacement (AVR) in nAF group were greater than those in AF group (58.2% vs. 4.3%, *p* < 0.001). Enlargement of left and right atrium might be associated with the increasing proportion of MVR + TVP in AF group.

**Table 1:** Characteristics of patients in the study.

	AF (*n* = 24)	nAF (*n* = 24)	*P* value
Gender (m/f)	10/14	9/15	0.768
Age (years)	55.33 ± 7.88	54.58 ± 11.53	0.794
BMI	23.25 ± 2.82	23.48 ± 3.42	0.806
NYHA class (n, I/II/III/IV)	0/3/20/1	0/3/18/3	0.541
White cell (*10^9/L)	5.66 ± 1.77	5.99 ± 1.51	0.492
Neutrophil (*10^9/L)	3.51 ± 1.39	3.75 ± 1.14	0.521
Lymphocyte (*10^9/L)	1.44 ± 0.58	1.53 ± 0.55	0.611
Blood glucose (mmol/l)	6.01 ± 1.46	5.88 ± 1.98	0.799
TG (mmol/l)	1.55 ± 0.74	1.83 ± 1.91	0.542
TC (mmol/l)	3.90 ± 0.97	4.45 ± 1.26	0.115
HDL (mmol/l)	0.90 ± 0.16	1.07 ± 0.26	0.013
LDL (mmol/l)	2.49 ± 0.73	2.72 ± 0.76	0.319
CRP (mg/l)	11.04 ± 19.61	17.86 ± 38.43	0.442
ESR (mm/h)	34.61 ± 27.69	27.73 ± 26.11	0.396
NTproBNP (pg/ml)	4118.52 ± 5309.2	3061.83 ± 2997.09	0.408
EF (%)	52.17 ± 11.42	54.75 ± 9.06	0.599
RVD (mm)	15.92 ± 4.62	16.63 ± 2.80	0.524
LVD (mm)	47.96 ± 10.39	48.21 ± 8.93	0.929
LAD (mm)	48.88 ± 8.34	38.50 ± 8.70	0.004
RAD (mm)	48.50 ± 9.82	43.00 ± 8.48	0.043
Smoking (n, %)	7 (29.17%)	5 (20.83%)	0.505
Drinking (n, %)	2 (8.33%)	4 (16.67%)	0.383
Hypertension (n, %)	5 (20.83%)	5 (20.83%)	1
CHD (n, %)	7 (29.17%)	3 (12.5%)	0.155
Diabetes (n, %)	3 (12.5%)	0 (0%)	0.074
Digitalis (n, %)	20 (83.33%)	15 (62.5%)	0.104
Amiodarone (n, %)	3 (12.5%)	0 (0%)	0.074
ACEI (n, %)	2 (8.33%)	2 (8.33%)	1
ARB (n, %)	1 (4.16%)	1 (4.16%)	1
β-blocker (n, %)	12 (50%)	17 (70.83%)	0.140
Ca^2+^ antagonist (n, %)	12 (50%)	9 (37.5%)	0.383
Statin (n, %)	3 (12.5%)	3 (12.5%)	1

Abbreviations used in table 1: *BMI* body mass index, *NYHA class* New York Heart Association class, *TG* triglyceride, *TC* total cholesterol, *HDL* high-density lipoprotein, *LDL* low-density lipoprotein, *CRP* C-reactive protein, *ESR* erythrocyte sedimentation rate, *NTproBNP* N-terminal pro B-type-natriuretic peptide, *EF* ejection fraction, *RVD* right ventricular dimension, *LVD* left ventricular dimension, *LAD* left atrial dimension, *RAD* right atrial dimension, *CHD* coronary heart disease, *ACEI* angiotensin converting enzyme inhibitors, *ARB* angiotensin receptor blocker

**Table 2:** Surgery of patients in the study

Surgery	AF (%)	nAF (%)
AVR	1 (4.3%)	14 (58.2%)
MVP	2 (8.7%)	0 (0)
MVR	2 (8.7%)	1 (4.3%)
MVP + TVP	1 (4.3%)	0 (0)
MVR + TVP	11 (47.9%)	3 (12.5%)
AVR + MVR	1 (4.3%)	3 (12.5%)
AVR + MVR + TVP	6 (21.8%)	3 (12.5%)

Abbreviations used in table 2: *AVR* aortic valve replacement, *MVP* mitral valvuloplasty, *MVR* mitral valve replacement, *TVP* tricuspid valvuloplasty

**Fig. 1:**
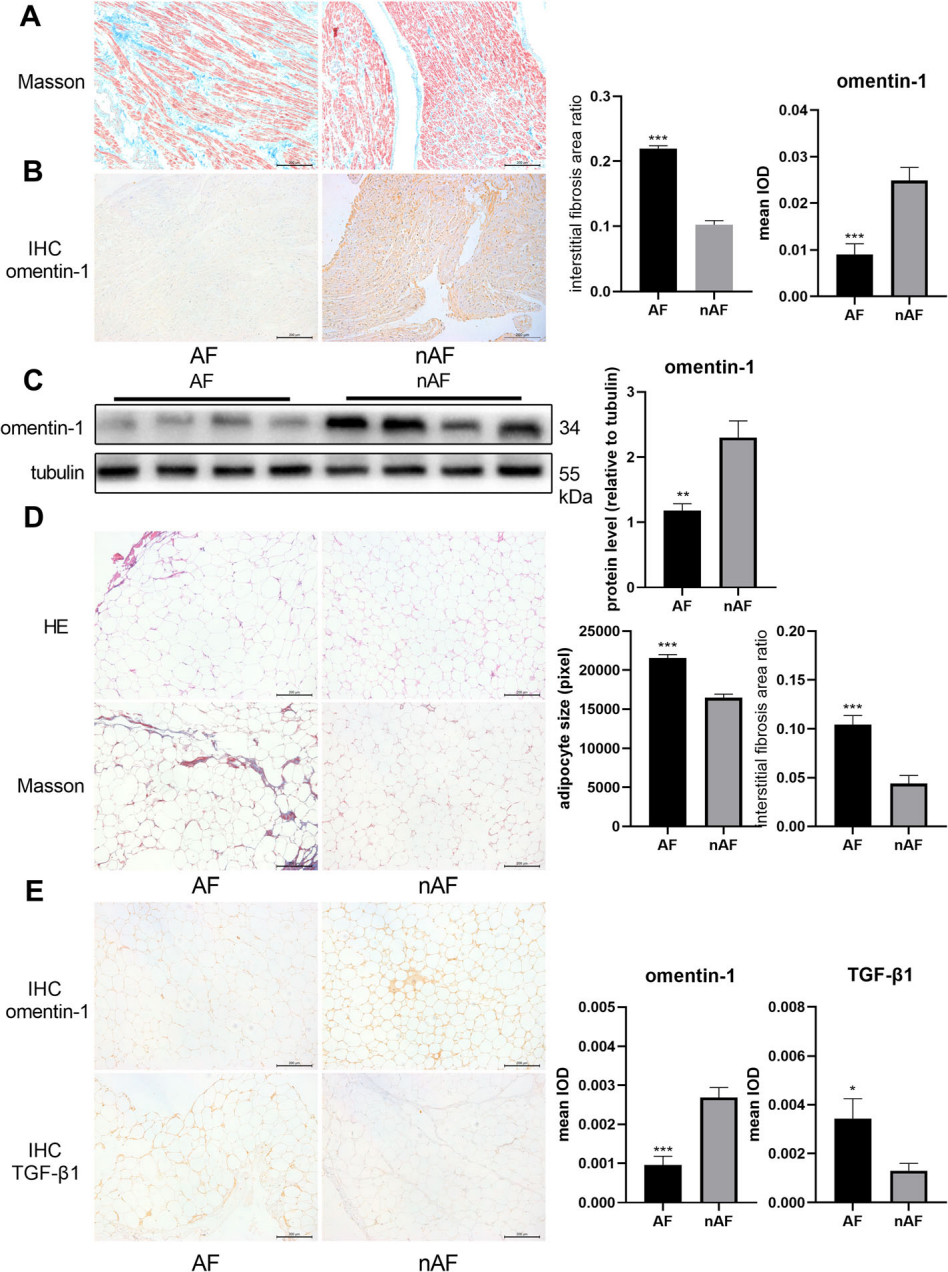
Epicardial adipose tissue (EAT) structure, omentin-1, and TGF-β1 expression in human samples. Representative pictures of Masson’s trichrome stained right atrial appendages (**a**) (100× magnification) revealed severe atrial fibrosis in patients with atrial fibrillation (AF) (AF group, *n* = 18; nAF group, *n* = 13). Representative immunohistochemical images of omentin-1 (**b**) in right atrial appendages and quantitative protein expression level. Omentin-1 expression in right atrial appendages was detected via western blotting (**c**) (AF group, *n* = 4; nAF group, *n* = 4). Representative images of H&E-stained EAT (**d**) (100× magnification) showed adipocyte hypertrophy in patients with AF (AF group, *n* = 20; nAF group, *n* = 20). Representative images of Masson’s trichrome-stained EAT (D) (100× magnification) indicated severe EAT fibrosis in patients with AF. Representative immunohistochemical images of omentin-1 (**e**) and TGF-β1 (**e**) in EAT and respective quantitative protein expression levels. *P < 0.05 vs nAF group, **P < 0.01 vs nAF group, ***P < 0.001 vs nAF group

In sections of right atrial appendages, the interstitial fibrosis area was significantly larger in the AF group (Fig. 1a), which was consistent with previous studies. The immunohistochemical assay and western blotting demonstrated that omentin-1 was downregulated in right atrial appendages of patients with AF (Fig. 1b, c). The mean adipocyte size was calculated as the ratio of total adipocyte size/adipocyte number. Analysis of the images of the H&E-stained sections revealed that the adipocytes of patients with AF were significantly larger than those of patients without AF (Fig. 1d). The interstitial fibrosis area ratio of EAT in the AF group was increased compared with that of the nAF group, as shown by Masson’s trichrome staining (Fig. 1d). The immunohistochemical assay revealed that omentin-1, which is a secretory protein, was expressed primarily in the ECM, and that its expression was decreased in the EAT from patients with AF (Fig. 1e). In contrast, the EAT from these patients exhibited high expression levels of TGF-β1 (Fig. 1e), a key mediator of structural remodeling.

### Omentin-1 inhibited TGF-β1-induced activation of CFs

**Fig. 2:**
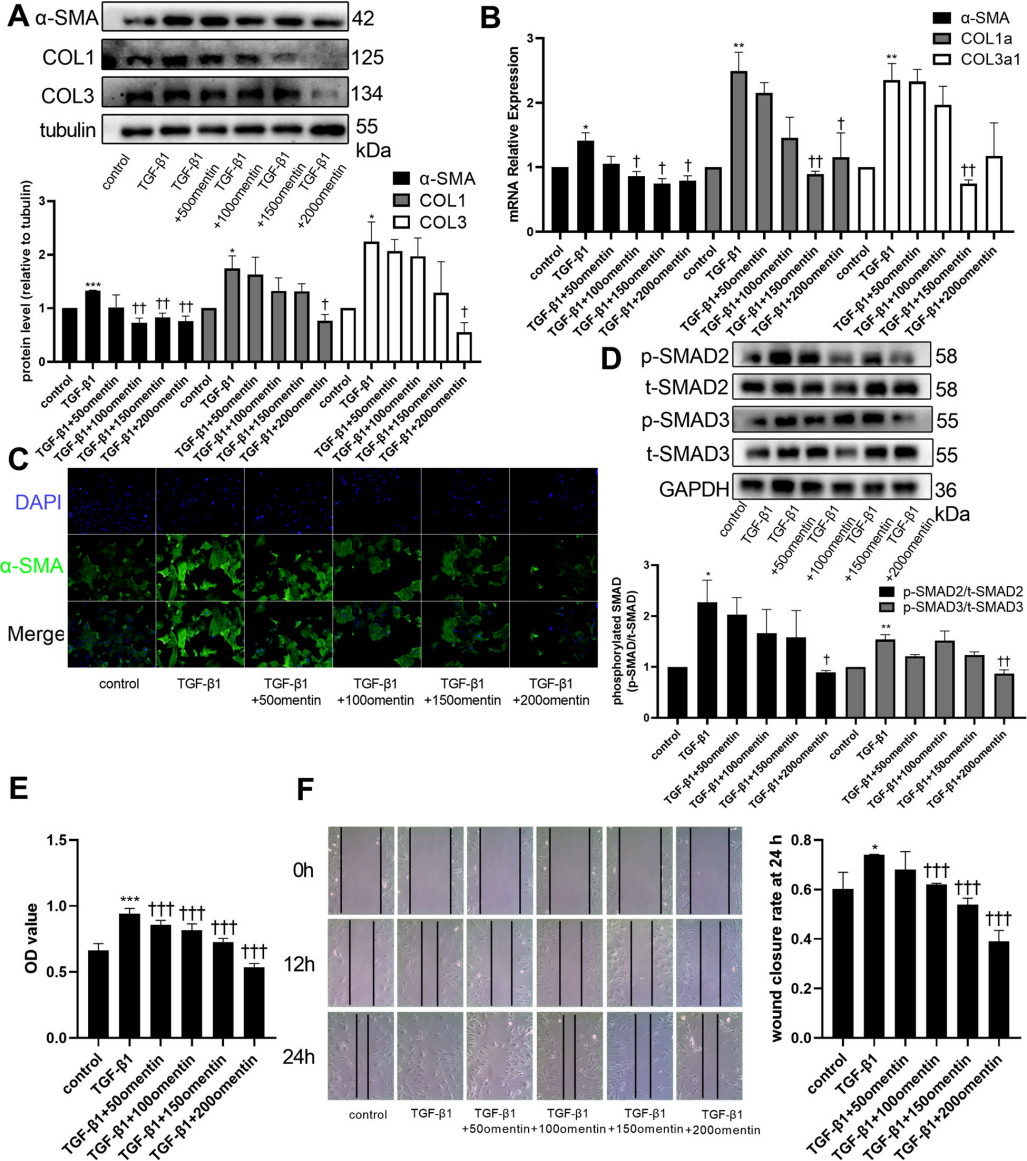
Omentin-1 inhibited TGF-β1-induced activation of cardiac fibroblasts (CFs). α-SMA, COL1, and COL3 protein levels in non-treated and treated CFs were detected via western blotting (**a**). The mRNA levels of α-SMA, COL1a, and COL3a1were detected via RT-qPCR. Expression of mRNA was normalized to GAPDH. (**b**) Representative images of immunofluorescence staining for α-SMA in CFs (**c**) (200× magnification) showed the antifibrotic effect of omentin-1. p-SMAD2, t-SMAD2, p-SMAD3, t-SMAD3 protein levels in CFs were detected via western blotting (**d**). (**e**) CF proliferation assay. (**f**) Representative images of the CFs scratch assay (100× magnification). The values were mean ± SEM of three independent experiments. *P < 0.05 vs control group, **P < 0.01 vs control group, ***P < 0.001 vs control group, †*P* < 0.05 vs TGF-β1 group, ††*P* < 0.01 vs TGF-β1 group, †††*P* < 0.001 vs TGF-β1 group

The increase in the expression levels of α-SMA, COL1, and COL3 induced by TGF-β1 in CFs was downregulated when cells were co-treated with omentin-1 at concentrations of 100–200 ng/mL (Fig. 2a). Omentin-1 treatment also attenuated TGF-β1-induced upregulation of α-SMA, COL1a and COL3a1 mRNA expression (Fig. 2b). The immunofluorescence studies demonstrated that omentin-1 attenuated TGF-β1-induced expression of α-SMA in a dose-dependent manner (Fig. 2c). TGF-β1 significantly activated the phosphorylation of SMAD2 and SMAD3, whereas omentin-1 at a concentration of 200 ng/mL inhibited the phosphorylation of SMAD2 and SMAD3 (Fig. 2d). The proliferation of CFs was greater when they were treated with TGF-β1 alone, whereas this capability was downregulated by simultaneous treatment with omentin-1 in a dose-dependent manner (Fig. 2e). The scratch assay demonstrated that omentin-1 also inhibited TGF-β1-induced cell migration in a dose-dependent manner (Fig. 2f). Together, these results suggested that omentin-1 inhibited TGF-β1-induced activation of CFs via the SMAD2/SMAD3 signaling pathway.

### Omentin-1 inhibited TGF-β1-induced EndMT of HUVECs

**Fig. 3:**
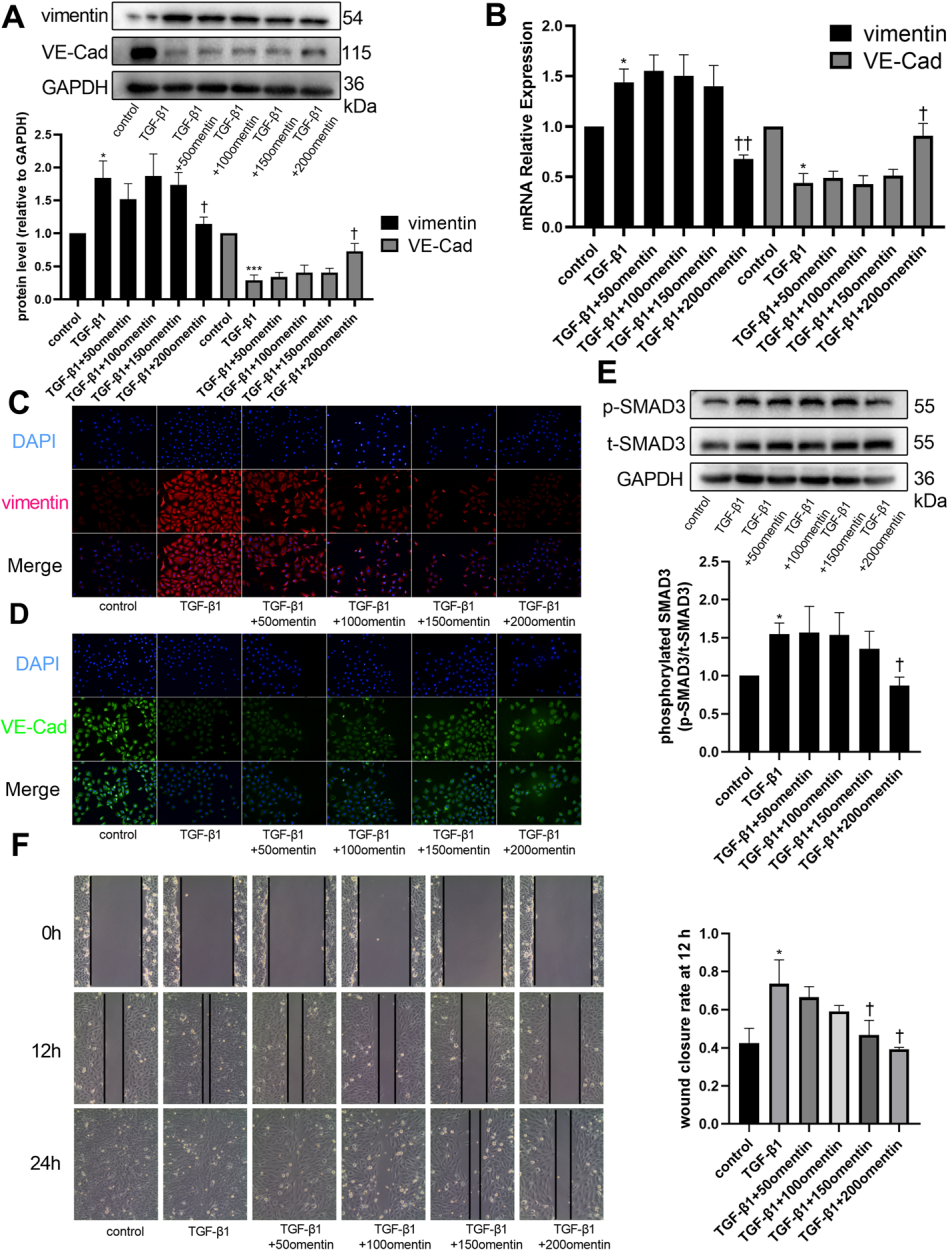
Omentin-1 inhibited TGF-β1-induced endothelial-mesenchymal transition (EndMT) of human umbilical vein endothelial cells (HUVECs). Vimentin and VE-Cad protein levels in HUVECs were detected via western blotting (**a**). The mRNA levels of vimentin and VE-Cad were detected via RT-qPCR (**b**). Expression of mRNA were normalized to GAPDH. Representative images of immunofluorescence staining for vimentin (**c**) and VE-Cad (**d**) (200× magnification) in HUVECs. The p-SMAD3 and t-SMAD3 protein levels in HUVECs were detected via western blotting (**e**). (**f**) Representative images of HUVECs scratch assay (× 100 magnification). The values were mean ± SEM of three independent experiments. *P < 0.05 vs control group, **P < 0.01 vs control group, ***P < 0.001 vs control group, †*P* < 0.05 vs TGF-β1 group, ††*P* < 0.01 vs TGF-β1 group, †††*P* < 0.001 vs TGF-β1 group

During structural remodeling, CFs are derived from many cell lineages; one important source of CFs are endothelial cells undergoing EndMT. HUVECs exhibited a high expression level of vimentin, a fibroblast marker, but lost their expression of VE-Cad, an endothelial cell marker, after treatment with TGF-β1 (Fig. 3a). Simultaneous treatment of HUVECs with TGF-β1 and omentin-1 (200 ng/mL) partly reversed the transition (Fig. 3a). mRNA expression level of vimentin increased, while that of VE-Cad decreased when the cells were treated with TGF-β1 alone, but omentin-1 ameliorated these changes at a concentration of 200 ng/mL (Fig. 3b). The immunofluorescence assay further validated the results and showed a dose-dependent effect (Fig. 3c, d). TGF-β1 activated the phosphorylation of SMAD3 in HUVECs, and this effect was also reduced by omentin-1 at a concentration of 200 ng/mL (Fig. 3e). However, the phosphorylation of SMAD2 in HUVECs remained unchanged when HUVECs were treated with TGF-β1 and/or omentin-1 (Supplementary Figure 1A). Upregulation of the migratory capability of endothelial cells is one of the features of EndMT. The scratch assay indicated that omentin-1 prevented TGF-β1-induced upregulation of HUVEC migration (Fig. 3f). In summary, these results revealed that omentin-1 partly reversed TGF-β1-induced EndMT of HUVECs via the SMAD3 signaling pathway.

### Hypoxia induced alterations in the secretion-phenotype of adipocytes

**Fig. 4:**
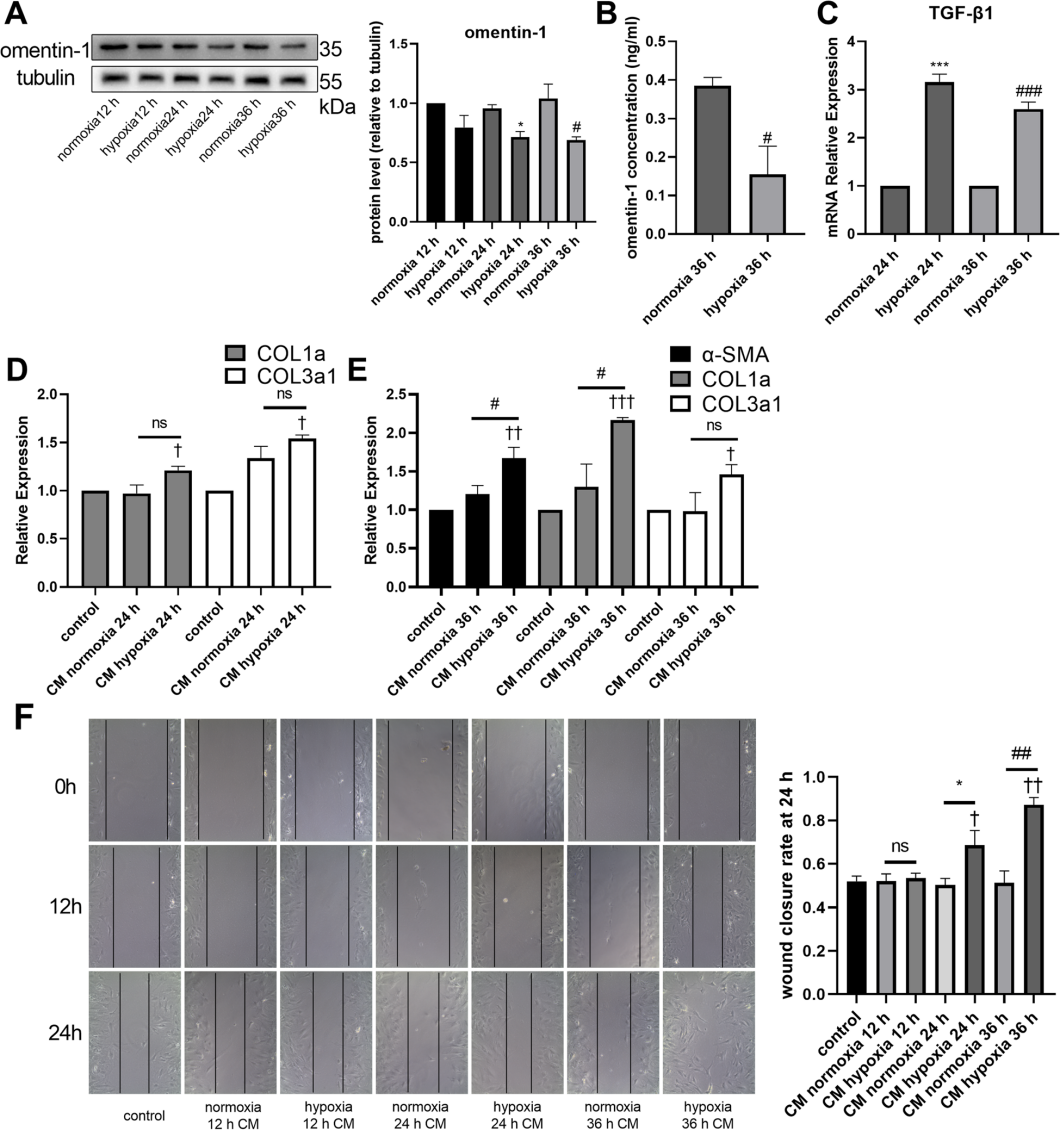
Hypoxia resulted in a change in the secretion phenotype of adipocytes. Omentin-1 protein levels in adipocytes was detected via western blotting (**a**). The concentrations of omentin-1 in the CM of adipocyte treated with normoxia or hypoxia were determined by ELISA (**b**). The mRNA levels of TGF-β1 in adipocytes (24 h normoxia, 24 h hypoxia, 36 h normoxia, 36 h hypoxia) was detected via RT-qPCR. Expression of mRNA was normalized to GAPDH. (**c**) The mRNA levels of α-SMA, COL1a, and COL3a1 in non-CM-treated or CM-treated CFs were detected via RT-qPCR. The mRNA level was normalized to GAPDH. (**d**, **e**) (**f**) Representative images of CFs scratch assay (100× magnification). The values were mean ± SEM of three independent experiments. *P < 0.05 vs normoxia 24 h group, ***P < 0.001 vs normoxia 24 h group, #*P* < 0.05 vs normoxia 36 h group, ###*P* < 0.001 vs normoxia 36 h group, †*P* < 0.05 vs control group, ††*P* < 0.01 vs control group, †††*P* < 0.001 vs control group

The larger adipocyte size and more severe interstitial fibrosis indicated that the adipocytes of patients with AF might have suffered from hypoxia. Therefore, the adipocytes were subjected to hypoxia (5% O_2_ and 95% N_2_) for 12, 24, and 36 h, and then the CM was collected for analysis. The omentin-1 protein level was significantly downregulated in the adipocytes after 24 and 36 h of hypoxia (Fig. 4a). However, no TGF-β1 expression in the normoxia and hypoxia groups was detected by western blotting (Supplementary Figure 1B). The omentin-1 concentration in the CM after 36 h of hypoxia was significantly downregulated compared with that in the CM after 36 h of normoxia (Fig. 4b). RT-qPCR revealed that the mRNA expression of TGF-β1 was increased in the 24 and 36 h hypoxia groups (Fig. 4c). Treatment with CM of the adipocytes did not affect the protein expression levels of α-SMA, COL1, and COL3 in CFs, or of vimentin and VE-Cad in HUVECs (Supplementary Figure 1C, D). Compared with the level in non-CM-treated CFs, the mRNA levels of COL1a and COL3a1 were increased in the group treated with the 24 h hypoxia CM, but there was no significant difference in the mRNA levels of COL1a and COL3a1 between the 24 h normoxia CM and 24 h hypoxia CM treatments (Fig. 4d). CFs treated with the 36 h hypoxia CM exhibited significantly higher mRNA levels of α-SMA, COL1a, and COL3a1 than the non-CM-treated cells, and the levels of α-SMA and COL1a in the 36 h hypoxia CM-treated cells were significantly upregulated comparing with the 36 h normoxia CM treated cells (Fig. 4e). The migratory capability of CFs was also enhanced after treatment with the hypoxia CM (Fig. 4f) while the migratory capability of HUVECs was not affected by the CM (Supplementary Figure 1E). The results revealed that hypoxia played an important role in changing the secretion-phenotype of adipocytes, with the CM exerting different effects on the CFs.

## Discussion

Structural remodeling, especially atrial fibrosis, is considered as a substrate of AF onset and progression, and thus could be a promising therapeutic target. Recently Haemers et al. reported that fibrotic remodeling of EAT was associated with AF in an obese sheep model [17]. In our previous study, we demonstrated that the level of EAT fibrosis was associated with EAT attenuation via computed tomography [18], and thus might be a more sensitive predictor of cardiovascular diseases than EAT volume [19]. Therefore, we designed this study to explore the relationship between EAT and atrial fibrosis in AF.

An analysis of the patients’ baseline data revealed that patients with AF had decreased HDL and increased RAD and LAD compared with patients without AF. Although HDL is a known protective factor in cardiovascular diseases, its association with AF remains unclear and controversial. Benjamin et al. reported that patients with AF had a normal lipid profile [20] whereas recently, Markus et al. demonstrated that alterations in HDL function were associated with AF [21]. Decreased HDL might be associated with AF and changes in the EAT secretion-phenotype, but further studies are needed to confirm this hypothesis. Higher RAD and LAD in patients with AF were consequences of structural remodeling and are associated with AF occurrence and development [22]. Consistent with previous studies [2], patients with AF in our study exhibited severe atrial fibrosis. Structural remodeling, particularly atrial fibrosis, is believed to be the substrate of AF initiation and progression. The remodeling involves cellular processes (e.g., CFs activation and cardiomyocyte apoptosis) and extracellular components (e.g., excessive ECM production). Under physiological conditions, cardiac ECM acts as a scaffold for cardiac tissue and mediates electrical conduction [23]. Homeostasis of ECM is mainly mediated by CFs. In healthy individuals, CFs remain in an inactive state and at a relative low population level [3]. However, under pathological situations, many profibrotic stimuli (e.g., TGF-β1, which is one of the most potent profibrotic cytokines) activate CFs, breaking ECM homeostasis and eventually leading to alterations of its quality and quantity [24]. TGF-β1 can induce the differentiation of CFs into myofibroblasts, which express α-SMA and have an approximately 2-fold higher ECM-producing capability than CFs [25]. TGF-β1 binds to TGF-β1 receptors (TβRI and TβRII) to induce SMAD2 and SMAD3 phosphorylation. Next the phosphorylated SMAD2 and SMAD3 translocate to the nucleus where they regulate the expression of fibrosis-related genes, including those encoding COL1 and COL3 [26], thereby resulting in atrial fibrosis. During atrial fibrosis, endothelial cells undergoing EndMT are a crucial source of fibroblasts, with TGF-β1-SMAD3 being the key signaling pathway mediating the process [27]. In our previous study, we had found that TGF-β1 was capable of inducing the expression of fibrotic markers in endothelial progenitor cells [28]. Notably omentin-1 was downregulated in patients with AF in this study, which propelled us to explore the roles of omentin-1 in atrial fibrosis. To study atrial fibrosis in vitro, we established models of TGF-β1-induced activation of CFs and TGF-β1-induced EndMT of HUVECs.

EAT is a secretory organ that undergoes changes in its structure and secretion phenotype in cardiovascular diseases. However, the exact cause of these changes is unclear. Volume is an important feature of adipose tissue (AT), and many researchers have tried to elucidate the relationship between EAT volume and cardiovascular diseases. The Framingham Heart Study involving 2317 participants showed that the volume of EAT was a predictor of AF risk [29]. Batal et al. revealed that left atrial fat thickness was associated with AF burden, independent of body mass index and left atrial area [30]. Adipocyte hypertrophy and/or hyperplasia leads to an expansion of AT. It is noteworthy that the size of adipocytes in patients with AF was increased, which meant that the EAT in these patients might undergo expansion. Excessively expanded AT suffers from hypoxia owing to the large cell size and insufficient neovasculature. The hypoxic microenvironment results in apoptosis and/or necrosis of adipocytes and infiltration of macrophages, which gather around the dead adipocytes and form crown-like structures [31]. Dead adipocytes and infiltration of macrophages initiate AT fibrosis. Adipocytes, preadipocytes and macrophages secrete collagens and other profibrotic factors and eventually lead to AT dysfunction [32]. The accumulation of ECM components induces more apoptosis and necrosis of adipocytes and infiltration of macrophages [33]. The EAT from patients with AF in this study showed severe interstitial fibrosis, the hallmark of AT dysfunction. Such dysfunction might alter its secretion phenotype and initiate and/or exacerbate atrial fibrosis. It has been reported that Activin A (a member of the TGF-β superfamily) secreted from EAT induced rat atrial fibrosis in an organ-culture model [34]. Since it was reported that serum omentin-1 concentration was inversely correlated with AF [14], we detected omentin-1 and TGF-β1 expression level in EAT and revealed that omentin-1 was downregulated, whereas TGF-β1 was upregulated in patients with AF. Considering the existence of paracrine signaling between EAT and the myocardium, the increased TGF-β1 expression in EAT might initiate and/or exacerbate atrial fibrosis in patients. However, the effect of omentin-1 in TGF-β1-induced atrial fibrosis is unknown. Therefore, models of TGF-β1-induced activation of CFs and TGF-β1-induced EndMT of HUVECs were used to explore roles of omentin-1 in these processes. The study demonstrated that omentin-1 inhibited TGF-β1-induced activation of CFs via the SMAD2/SMAD3 signaling pathway. Also, TGF-β1 activated the phosphorylation of SMAD3, upregulated the expression level of vimentin while downregulated the expression level of VE-Cad in HUVECs indicating that HUVECs underwent EndMT when exposed to TGF-β1. However, omentin-1 at the concentration of 200 ng/ml can reduce phosphorylation level of SMAD3, restore expression levels of vimentin and VE-Cad, block TGF-β1/SMAD3 signaling pathway, and prevent EndMT in HUVECs. These results suggested that omentin-1 acted as an antifibrotic factor, counteracting the effects of TGF-β1. The balance between profibrotic and antifibrotic factors in EAT may be important for the atrial myocardium to resist fibrotic changes and onset of AF.

The balance between profibrotic and antifibrotic factors mentioned above is disrupted in case of EAT dysfunction. As the early stage of AT dysfunction involves a hypoxic microenvironment, we induced hypoxia in adipocytes to evaluate their secretion phenotype and the effects of their CM on CFs and HUVECs. Western blot analysis revealed that the omentin-1 protein level in adipocytes was significantly reduced after 24 and 36 h of hypoxia, and RT-qPCR demonstrated that the TGF-β1 mRNA level was upregulated under those conditions. Additionally, omentin-1 concentration was significantly decreased in the CM of adipocytes with 36 h hypoxia treatment compared with that in the CM of adipocytes with 36 h normoxia treatment. However, we did not detect any expression of TGF-β1, COL1 and COL3 in adipocytes via western blotting. We hypothesized that adipocytes might not have been the main source of these fibrosis-related proteins, or because the sensitivity of the method used was too low to detect these proteins. Azizah et al. reported that hypoxia induced upregulation of IL-6 and leptin while inhibiting adiponectin secretion [35]. Our findings were similar to those of Azizah et al. to some extent, in that hypoxia alone could be the early trigger for altering the secretion phenotype of adipocytes and breaking the balance between profibrotic and antifibrotic adipocytokines. Therefore, EAT in patients with AF might undergo a change in their secretion phenotype at an early stage of hypoxia. Next, CFs and HUVECs were treated with the CM of adipocytes to determine whether they were also affected by the hypoxia-induced change of the secretion phenotype. The results revealed that CFs were activated when treated with CM of adipocytes cultured under hypoxia for 24 and 36 h. The phenomenon meant that EAT might initiate and/or exacerbate atrial fibrosis even in the early stage of dysfunction.

EAT is a promising therapeutic target owing to its specific anatomy and paracrine effects. However, the exact mechanism through which it mediates cardiovascular diseases is still unknown. Limited human EAT samples and few applicable animal models hinder research on EAT. Our study suggested that relieving the hypoxic microenvironment of EAT may help in maintaining the balance between profibrotic and antifibrotic factors and thus reduce atrial fibrosis. Further research on the signaling pathways that mediate the changes in the EAT secretion phenotype are needed.

## Conclusion

Our research showed that in patients with AF, EAT had larger adipocytes and higher interstitial fibrosis area ratios. Moreover, the omentin-1 level was reduced in EAT and right atrial appendages whereas TGF-β1 was upregulated in EAT. Omentin-1 prevented TGF-β1-induced activation CFs and TGF-β1-induced EndMT of HUVECs. We also found that hypoxia was an important factor that mediated changes in the secretion phenotype of adipocytes.

## Supplementary information

**Additional file 1: Table S1.** Primes used in the article.

**Additional file 2: Figure S1.** The p-SMAD3 and t-SMAD3 protein levels in HUVECs treated with the CM of adipocytes were detected via western blotting (A) (n=3). TGF-β1, COL1, and COL3 protein levels in adipocytes treated with normoxia or hypoxia were detected via western blotting (B) (n=3). α-SMA, COL1, and COL3 protein levels in CFs treated with the CM of adipocytes were detected via western blotting (C) (n=3). Vimentin and VE-Cad protein levels in HUVECs treated with the CM of adipocytes were detected via western blotting (D) (n=3). (E) Representative scratch assay images of HUVECs treated with the CM of adipocytes (×100 magnification).

## Data Availability

All data generated or analyzed during this study are included in this published article and its supplementary information files.

## Abbreviations

EAT: Epicardial adipose tissue
AF: Atrial fibrillation
H&E: Hematoxylin and eosin
CFs: Cardiac fibroblasts
EndMT: Endothelial-mesenchymal transition
HUVEC: Human umbilical vein endothelial cell
ECM: Extracellular matrix
α-SMA: Alpha smooth muscle actin
COL1: Collagen-1
COL3: Collagen-3
IL: Interleukin
RANK: Receptor activator of NF-κB
TGF-β1: Transforming growth factor beta 1
FBS: Fetal bovine serum
DMEM: Dulbecco’s modified Eagle’s medium
RT-qPCR: Quantitative reverse transcription-polymerase chain reaction
ELISA: Enzyme-linked immunosorbent assay
BMI: Body mass index
NYHA class: New York heart association class
TG: Triglyceride
TC: Total cholesterol
HDL: High-density lipoprotein
LDL: Low-density lipoprotein
CRP: C-reactive protein
ESR: Erythrocyte sedimentation rate
NTproBNP: N-terminal pro B-type-natriuretic peptide
EF: Ejection fraction
RVD: Right ventricular dimension
LVD: Left ventricular dimension
LAD: Left atrial dimension
RAD: Right atrial dimension
CHD: Coronary heart disease
ACEI: Angiotensin converting enzyme inhibitors
ARB: Angiotensin receptor blocker
CM: Conditioned medium
AT: Adipose tissue
AVR: Aortic valve replacement
MVP: Mitral valvuloplasty
MVR: Mitral valve replacement
TVP: Tricuspid valvuloplasty
