# Supplementary material for: Omentin-1 is associated with atrial fibrillation in patients with cardiac valve disease
Source: BMC Cardiovasc Disord. 2020 May 6;20:214. doi: 10.1186/s12872-020-01478-1 (PMC7203903; doi:10.1186/s12872-020-01478-1)
Supplement: Supplementary file 1 — Additional file 1: Table S1. Primes used in the article. [file 12872_2020_1478_MOESM1_ESM.docx]

Supplementary File

Supplementary Table 1

Primes used in the article

| human GAPDH | forward | 5’ CCAGCAAGAGCACAAGAGGAAGAG 3’ |
| --- | --- | --- |
|  | reverse | 5’ GGTCTACATGGCAACTGTGAGGAG 3’ |
| rat GAPDH | forward | 5’ ACGGCAAGTTCAACGGCACAG 3’ |
|  | reverse | 5’ CGACATACTCAGCACCAGCATCAC 3’ |
| mouse GAPDH | forward | 5’ AAGAAGGTGGTGAAGCAGGCATC 3’ |
|  | reverse | 5’ CGGCATCGAAGGTGGAAGAGTG 3’ |
| Vimentin | forward | 5’ TTGCCGTTGAAGCTGCTAACTACC 3’ |
|  | reverse | 5’ AATCCTGCTCTCCTCGCCTTCC 3’ |
| VE-Cad | forward | 5’ CTACGATGTGTCGGTGCTCAACTC 3’ |
|  | reverse | 5’ TGGTCCGCCTCGTCCTTCTTC 3’ |
| α-SMA | forward | 5’ GCGTGGCTATTCCTTCGTGACTAC 3’ |
|  | reverse | 5’ CCATCAGGCAGTTCGTAGCTCTTC 3’ |
| COL1a | forward | 5’ TGTTGGTCCTGCTGGCAAGAATG 3’ |
|  | reverse | 5’ GTCACCTTGTTCGCCTGTCTCAC 3’ |
| COL3a1 | forward | 5’ GACACGCTGGTGCTCAAGGAC 3’ |
|  | reverse | 5’ GTTCGCCTGAAGGACCTCGTTG 3’ |
| TGF-β1 | forward | 5’ GCAACAATTCCTGGCGTTACCTTG 3’ |
|  | reverse | 5’ CAGCCACTGCCGTACAACTCC 3’ |
