# Supplementary material for: Omentin-1 is associated with atrial fibrillation in patients with cardiac valve disease
Source: BMC Cardiovasc Disord. 2020 May 6;20:214. doi: 10.1186/s12872-020-01478-1 (PMC7203903; doi:10.1186/s12872-020-01478-1)
Supplement: Supplementary file 2 — Additional file 2: Figure S1. The p-SMAD3 and t-SMAD3 protein levels in HUVECs treated with the CM of adipocytes were detected via western blotting (A) (n=3). TGF-β1, COL1, and COL3 protein levels in adipocytes treated with normoxia or hypoxia were detected via western blotting (B) (n=3). α-SMA, COL1, and COL3 protein levels in CFs treated with the CM of adipocytes were detected via western blotting (C) (n=3). Vimentin and VE-Cad protein levels in HUVECs treated with the CM of adipocytes were detected via western blotting (D) (n=3). (E) Representative scratch assay images of HUVECs treated with the CM of adipocytes (×100 magnification). [file 12872_2020_1478_MOESM2_ESM.docx]

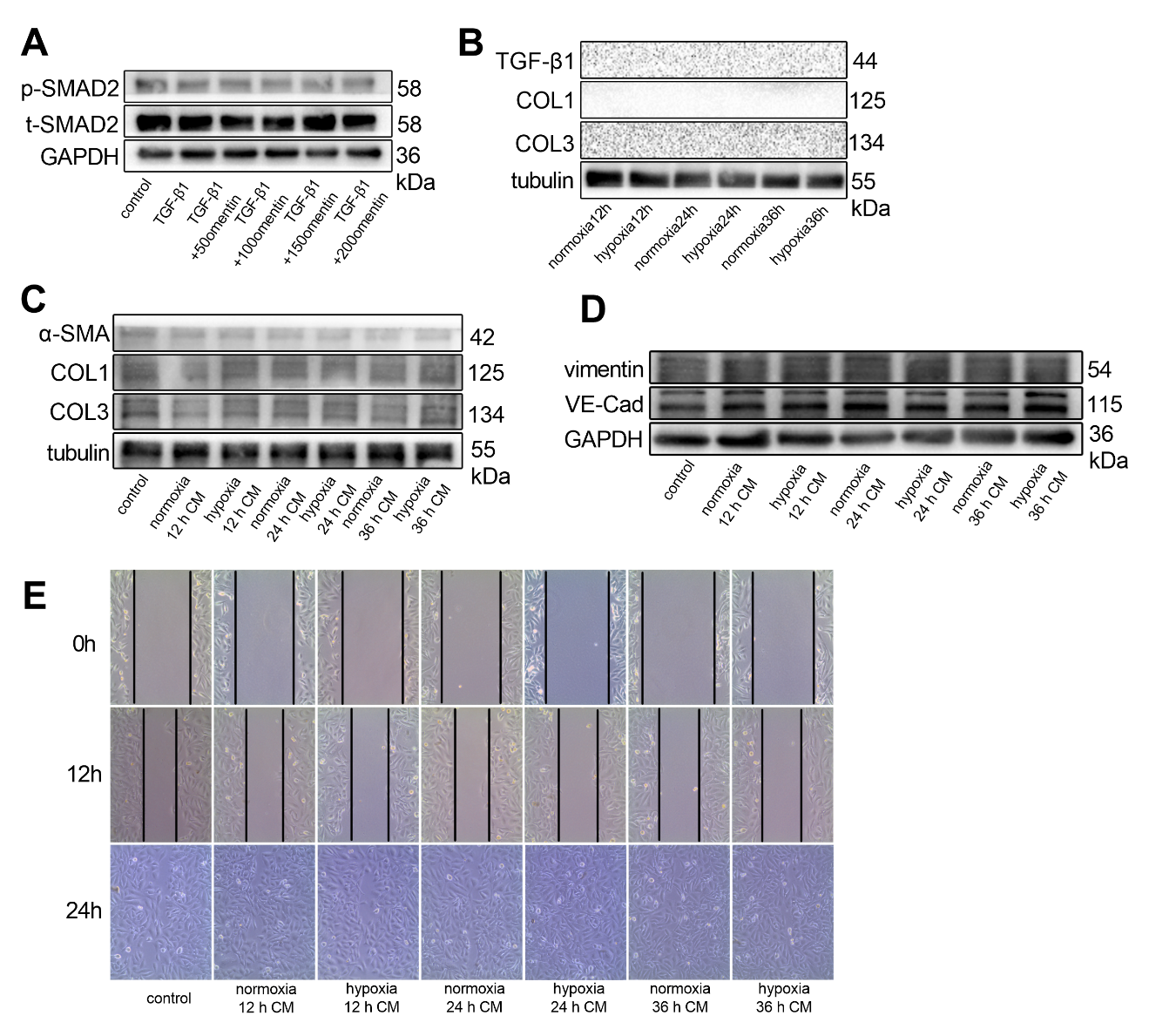


Supplementary figure 1: The p-SMAD3 and t-SMAD3 protein levels in HUVECs treated with the CM of adipocytes were detected via western blotting (A) (n=3). TGF-β1, COL1, and COL3 protein levels in adipocytes treated with normoxia or hypoxia were detected via western blotting (B) (n=3). α-SMA, COL1, and COL3 protein levels in CFs treated with the CM of adipocytes were detected via western blotting (C) (n=3). Vimentin and VE-Cad protein levels in HUVECs treated with the CM of adipocytes were detected via western blotting (D) (n=3). (E) Representative scratch assay images of HUVECs treated with the CM of adipocytes (×100 magnification).
